# Cultural tightness and scientific capacity: A cross-national study of their synergistic and conflicting roles in COVID-19 pandemic outcomes

**DOI:** 10.1371/journal.pone.0330983

**Published:** 2026-07-30

**Authors:** Chris William Callaghan

**Affiliations:** School of Economics, Finance and Law, Anglia Ruskin University, Chelmsford, United Kingdom; University of Cagliari: Universita degli Studi Di Cagliari, ITALY

## Abstract

Despite their advanced scientific capacity, many nations experienced high COVID-19 cases and deaths during the pre-vaccine phase of the pandemic. In contrast, some countries with tighter cultural norms, where social behaviour is more strongly regulated, appear to have controlled the spread of the virus more effectively. This creates a paradox: the same norm strength that may support compliance with public-health measures may also be less conducive to ideation, creativity, and scientific output. While prior research has associated cultural tightness with lower COVID-19 cases and deaths, its relationship with scientific capacity remains underexplored. This study examines associations between scientific capacity, cultural tightness, and COVID-19 outcomes using cross-national data. Findings show that scientific capacity has no robust direct protective association with pre-vaccine COVID-19 cases or deaths once cultural tightness and structural covariates are included. In contrast, Gelfand’s measure of cultural tightness is consistently associated with lower cases and deaths. These findings suggest that scientific knowledge alone does not automatically translate into population-level protection, and that norm strength, behavioural coordination, and knowledge-to-action capacity may be important conditions of pandemic resilience.

## 1. Introduction

To what extent did a nation’s scientific expertise shield its population from the impacts of the COVID-19 pandemic? Paradoxically, countries recognized as global leaders in scientific research saw some of the highest rates of cases and deaths. This counterintuitive phenomenon presents a compelling challenge, raising critical questions about the relationship between scientific capacity and pandemic- and disaster- resilience. Interestingly, recent findings suggest that countries such as China with tight cultural norms were more effective at reducing COVID-19 cases and deaths in the pre-vaccine phase of the pandemic [1,2]. Cultural tightness refers to differences between societies in “strength of social norms and tolerance of deviant behaviour” [3]. Drawing on tightness–looseness theory, Gelfand argued early in the pandemic that the culturally loose United States needed to “tighten up” to improve its response to COVID-19 [4].

Despite growing evidence that countries with tighter cultural norms experienced better disaster outcomes, including lower COVID-19 cases and deaths [1,2,4], and despite well-developed theoretical explanations for why tightness may improve collective response under threat so [3,5–7], less attention has been given to a potential drawback of tight cultures, namely their possible constraint on innovation and scientific progress [8]. Although endogenous growth and recombinant innovation theories do not directly theorise cultural tightness, they suggest that knowledge production depends partly on creativity, experimentation, recombination, and the exploration of novel ideas [9–11]. To the extent that tight cultures constrain behavioural variation and reduce tolerance for deviant or unconventional approaches, they may therefore be less conducive to some forms of innovation and scientific output.

This tension is important because cultural norms and values are widely understood to shape a range of innovation-related processes, including knowledge creation, technological change, innovation performance, technology adoption, and innovation diffusion [12–20].

Norms and values that restrict behavioural variability have been associated with lower creative or innovative activity [8] and societal values such as uncertainty avoidance, collectivism, and future orientation have been found to correlate negatively with measures of scientific innovativeness, including university-industry technological transfer and the contribution of research to economic and technological development [21]. A paradox therefore arises, whereby the same tightness-related norms that may reduce COVID-19 cases and deaths by increasing compliance with mask wearing, lockdowns, stay-at-home orders, and other non-pharmaceutical interventions may also constrain the cultural conditions under which scientific capacity is generated or translated into effective response. It is therefore unclear whether tightness complements or weakens the contribution of scientific capacity to pandemic outcomes.

Given evidence that tight cultures are associated with reduced disaster consequences generally [3], and lower COVID-19 cases and deaths specifically [1,2,4], an important theoretical question remains concerning the relationship between cultural tightness and scientific capacity in pandemic response. Tightness may support the implementation of public health guidance by increasing behavioural conformity, yet it may also constrain some of the variation, experimentation, and openness associated with scientific and innovative activity. The work therefore tests whether the protective association between scientific capacity and COVID-19 cases and deaths is weaker in countries with higher cultural tightness. In doing so, the paper makes the following contributions.

First, it extends literature on scientific capacity to consider important and under-studied moderation effects of country culture on contributions of scientific capacity to disaster response. A failure in the literature to do this may have consequences, contributing to a lack of life-saving knowledge of relevant future possible disasters. The work here seeks to address this deficiency, thereby contributing to scientometric literature concerned with evaluating the effectiveness of scientific research [22–30], particularly regarding the COVID pandemic [31,32].

Second, the paper contributes to linking scientometric literature to recent debates in the cross-cultural management literature concerning the value of tightness values. Early successes of China in COVID response [33] contrast markedly from that of some democracies, including the United States (U.S.) [34]. Little is known, however, of the specifics of how societal culture affects COVID response. Culture is defined by Hofstede as “collective programming of the mind that distinguishes the members of one group or category of people from others” [35] and by the Global Leadership and Organizational Behavior Effectiveness (GLOBE) studies as “shared motives, values, beliefs, identities, and interpretations or meanings of significant events that result from common experiences and members of collectives and that are transmitted across age generations” [8].

Given the breadth of these definitions, the work here extends the analysis beyond hypothesis tests based on Gelfand et al.’s (2011) cultural tightness indicator. For descriptive comparison, it also examines Uz’s (2015) looseness indicator of cultural norm homogeneity, Hofstede’s (2011) indulgence/restraint and individualism constructs, and the GLOBE in-group collectivism measure (House et al., 2004). In doing so, it links the scientometric and empirical cultural literatures with a relatively complete coverage of theory, providing more of a ‘full house’ of tests of current theoretical models.

In summary, cultural tightness/looseness perspectives suggest a single cultural *norm*- tightness- has important causal effects on country-level outcomes [3,7,36]. In contrast, the Hofstede (2011) and GLOBE [8] perspectives are more comprehensive in that they claim their dimensions summarise all human societal *values*. The Hofstede and GLOBE schools derive their theoretical dimensions inductively using factor analysis and rigorous validity testing. In including both tightness norms as well as three values items that theoretically overlap with tightness, the work contributes by testing more nuanced theoretical predictions about specific channels through which tighter values might affect contributions of scientific capacity to reducing pandemic outcomes. By testing interactions between scientific capacity and each of these cultural indicators, the work examines whether the association between scientific capacity and COVID-19 outcomes varies across different cultural mechanisms.

Finally, this paper contributes to literature on innovation and scientific capacity to investigate a paradox associated with a seeming failure of scientific capacity to adequately protect countries from Covid cases and deaths in the pre-vaccine phase of the pandemic. The United States [34], with its extensive scientific capacity, together with some other world-leaders in scientific capacity, were amongst the worst affected by the pandemic in this phase. This is paradoxical, as scientific capacity should improve pandemic response in multiple ways, even in the short term, in that it is expected to provide and disseminate much needed (lifesaving) new and disruptive ideas [37]. The paper proceeds as follows. First, the relevant theory and literature are reviewed. The methodology and empirical approach are then described, followed by the presentation of results. The findings are then discussed, and the paper concludes by outlining limitations and recommendations for further research.

## 2. Theory and literature

Scientific capacity can aid pandemic response by informing life-saving policy measures. Ferguson et al.’s [38] model, for example, suggests suppression of the virus is preferable to mitigation, or allowing the virus to spread within the population to develop ‘herd’ immunity. The extent to which countries share ideas, however, is limited and local scientific capacity is necessary to derive localized practices from global knowledge. Local scientific capacity is vital to apply knowledge in line with contextual conditions. Patterns of cross-country collaborative and network linkages associated with scientific activity [39–43] may act as channels through which knowledge about pandemic response can be collaboratively shared, a knowledge collaboration effect. Scientific activity is a key component of a country’s innovative capability [44], contributing to the dynamics of its national innovation system [45–52] and its national innovative capacity [53]. A country with greater scientific capacity is expected to be better able to access (and appropriately apply to its own context) valuable global knowledge of best practices regarding reducing pandemic cases and deaths. Specific mechanisms have been identified that suggest how ideas, and ideation, coming up with ideas, translate into real world outcomes. For example, Romer’s (1990) production function can be read as a theory of how codified scientific knowledge scales human survival. Once discovered, a life-saving idea is non-rival, in that its use is not depleted by repetition, and its social return can vastly exceed its initial cost of discovery. Oral rehydration therapy illustrates this mechanism [54]. The scientific insight that a simple combination of water, sugar, and salts can prevent death from diarrhoeal disease is inexpensive to reproduce, can be combined with basic local inputs, and can be deployed across large populations, especially in resource-constrained settings. Scientific ideas can thus reduce deaths at scale not only through frontier technologies, but through low-cost process innovations and behaviourally actionable protocols that improve survival. Applied to COVID-19, this same logic suggests that scientific capacity should reduce cases and deaths through the generation, validation, and diffusion of treatments, public-health protocols, and behavioural interventions. However, Jones cautions that the realised effect of scientific capacity may be weaker than Romer’s [55] logic alone would imply, because knowledge production is also constrained by duplication, congestion, and absorptive limits [56]. COVID-19 therefore provides a useful empirical setting in which to test how strongly the mortality-reducing potential of scientific capacity survives these real-world frictions.

Although the present analysis does not separately identify each transmission channel, the mechanisms through which ideas, and particularly scientific ideas, translate into real-world outcomes are well established in the endogenous growth literature and work that extends it [10,11]. In this view, codified knowledge enlarges the feasible set of interventions, improves the productivity of problem-solving, lowers the marginal cost of replication and diffusion, and enables cumulative adaptation in diagnostics, treatment protocols, behavioural guidance, and public-health delivery. The present analysis therefore does not test each mechanism in isolation; rather, it tests whether the aggregate stock of scientific knowledge, proxied here by the logged value of technical scientific outputs, is associated with fewer COVID-19 cases and deaths in a manner consistent with the Romer-Jones knowledge accumulation framework. Hypothesis A is therefore derived, *that scientific capacity is directly negatively associated with cases*, as well as Hypothesis B, *that scientific capacity is directly negatively associated with deaths*.

As discussed, the importance of testing these hypothesized associations is demonstrated by countries like the United States, which, paradoxically, despite its scientific leadership, experienced disproportionately high numbers of COVID-19 cases and deaths. Although the Romer-Jones framework explains how accumulated scientific knowledge may reduce COVID-19 cases and deaths, it does not explicitly incorporate cultural tightness-looseness; accordingly, tightness is modelled here as a distinct contextual mechanism, including through its interaction with scientific output.

Asymptomatic COVID-19 cases are also transmissible. Effectiveness of social distancing therefore depends on proportions of cases with mild symptoms and a propensity of individuals to self-isolate effectively- the extent to which those who are non-symptomatic follow scientific health guidelines [57]. Populations in tighter cultures may be more likely to follow scientific guidelines to the letter, and to stay at home and isolate even if asymptomatic. Asymptomatic cases at the time comprised between 20% and as 85% of total tested cases; testing is therefore important, but widespread shortages of tests and their components persisted globally [58]. Indeed, how “individuals respond to advice on how best to prevent transmission” would be “as important as government actions, if not more important” [57]. As discussed, individuals in tighter cultures may therefore be more inclined to comply with NPIs and to follow guidelines to seek testing. Models suggest NPIs such as lockdowns may be effective but when relaxed cases and deaths can start to once again increase rapidly [59]. One might expect tightness values to be negatively associated with COVID-19 cases and deaths, as per previous research [1,2] and the same hypotheses is therefore tested with our data which provides almost complete coverage of the pre-vaccine period, Hypothesis C, that *tightness is directly negatively associated with cases*, and Hypothesis D, that *tightness is directly and negatively associated with deaths*.

In testing these hypotheses, the tightness (Gelfand et al., 2011) index is used, as it theoretically represents the cultural norm of tightness, and tests are performed for further insight, using a looseness (Uz, 2015) index, a measure of cultural homogeneity of a country. As discussed, Hofstede’s (2011) indulgence and individualism values and the GLOBE (House et al., 2004) collectivism (in-group collectivism) values indices are also used to test further relevant theoretical predictions of cultural values theory. These values are expected to affect COVID response through the tightness channel in that they overlap theoretically with tightness even though they each differ in the mechanisms through which they might affect a society’s pandemic response. In that each of the cultural values orientations is (theoretically) expected to act differently than others, these tests provide a more specific and nuanced perspective of the ways in which they overlap with tightness norms.

### Interactions of scientific capacity and cultural tightness

Cultural values can affect transfers of knowledge, particularly across borders, and can influence a country’s absorptive capacity [60]. Cultural differences can, therefore, increase or decrease transaction costs of knowledge transfer and affect the likelihood of sharing and applying new knowledge. Cultural differences may reflect preferences for organized or formal (versus informal) and unstructured (versus structured) processes of communication [60], which may matter for pandemic communication efforts. Absorptive capacity depends on stocks of prior related knowledge, but knowledge creation is path-dependent. Differences in national cultures can therefore influence “dynamics and outcomes of cross-border knowledge transfer” [60]. Cross-country testing is, therefore, appropriate, in that policy differences in scientific capacity are taken into account in the analysis.

The primary channel through which tightness reduces pandemic outcomes may be through increasing the effectiveness of NPIs. History offers some insights into effectiveness of NPIs. Cities that intervened earlier and more aggressively in their use of NPIs such as lockdowns seemed to grow faster after the 1918 pandemic [61]. Populations exhibiting less variation in their societal norms may be more likely to conform to NPIs such as lockdowns and mask wearing. Policy makers might be mistaken if they recommend tightening of values as a solution to pandemic threats without considering if such tightening has other costs.

Although other studies suggest that cultural norms and values can affect innovativeness [12–20], and although these studies highlight different mechanisms through which culture can affect innovativeness they do not specifically consider tightness. Nevertheless, through mechanisms associated with restricting variability in human behaviour tightness norms can reduce individual creativity required for scientific activity. Uncertainty avoidance, collectivism, and future orientation are values measures that restrict this variability and they have been found to correlate negatively with a measure of scientific activity [8]. The final hypotheses are therefore derived: Hypothesis E, *that cultural tightness weakens the protective association between scientific capacity and cases*, and Hypothesis F, *that cultural tightness weakens the protective association between scientific capacity and deaths*. Further tests are conducted using an additional measure of cultural looseness and selected cultural value dimensions. Although not formally hypothesized, these analyses provide a broader descriptive assessment of whether related cultural mechanisms correspond with the main tightness–looseness findings. They also examine whether these dimensions are associated with COVID-19 cases and deaths directly, or indirectly through interactions with scientific capacity. The next sections therefore briefly review the literature on each value dimension to clarify the expected direction of these exploratory tests.

#### Indulgence versus restraint.

Hofstede [35] incorporates Minkov’s [62] work to derive a sixth culture dimension of his comprehensive framework, namely indulgence versus restraint. In response to the pandemic, Hofstede (2020) suggested certain theoretical channels through which indulgence and individualism affect Covid outcomes. Countries higher in indulgence may differ in pandemic response from restrained societies in that the latter may have greater tolerance of misery and hardship [63]. For Hofstede [35], an indulgent society is one “that allows relatively free gratification of basic and natural human desires related to enjoying life and having fun” and restraint characterises a society “that controls gratification of needs and regulates it by means of strict social norms.” Indulgent societies are expected to be at a greater risk of non-compliance with NPIs than restrained societies. For many of the reasons previously discussed one might expect more restrained societies to have significantly lower cases and deaths but to also have weaker contributions of scientific capacity to the same outcomes. Indulgence versus restraint may be a primary channel or mechanism through which values and norms affect COVID-19 cases and deaths. If so, then one would expect tests of interactions of scientific capacity and both tightness norms and restraint values to be similar in their associations with cases and deaths.

#### Individualism/Collectivism.

Hofstede [35] defines individualism versus collectivism as “the degree to which people in a society are integrated into groups”. According to Hofstede (2011, p. 3), the “most common dimension used for ordering societies is their degree of economic evolution or modernity”. The GLOBE studies split Hofstede’s collectivism construct, defining in-group collectivism as the degree “to which a culture’s people (should) take pride in and (should) feel loyalty toward their families, organizations, and employers”, and institutional collectivism as the degree “to which individuals are (should be) encouraged by institutions to be integrated into broader entities with harmony and cooperation as paramount principles at the expense of autonomy and individual freedom” [60].

Hofstede (2020:1) suggests that individualism rather than collectivism might enable COVID response in that society “could respond very quickly to a change in external factors” (Hofstede, 2020:1). If so, then Hofstede’s individualism measure would be expected to act synergistically with scientific capacity in that it could improve responsive uptake of scientific information. Tests of interactions between scientific capacity and each cultural measure, including tightness norms, looseness norms, and theoretically related cultural values, would therefore help to indicate whether these cultural mechanisms complement or constrain the effects of scientific capacity. Both societal response and scientific capability are likely to be central to pandemic control, but during the pre-vaccination phase outcomes may have depended primarily on the consistent and effective implementation of non-pharmaceutical interventions (NPIs).

Both institutional and in-group collectivism have also been found to be negatively associated with success in basic science [8], also highlighting the potential for countervailing effects of tight values such as collectivism and scientific capacity on COVID-19 response. One might therefore expect collectivism to enable conformity to NPIs and thus reduce COVID-19 cases and deaths, but also expect it to constrain innovative activity (which is primarily individualistic). Collectivism (individualism) is therefore expected to associate negatively (positively) with COVID-19 cases and deaths but to reduce (strengthen) the strength of associations between scientific capacity and the same outcomes. Interaction tests are expected to reveal patterns of associations of scientific capacity with cases and deaths.

Certain covariates are included in the regression-based test to partial out some confounding variance. Per capita GDP is included to control for country differences in income, and fixed capital formation to control for differences in capital infrastructure. Countries with larger capital investments might have fewer cases due to better infrastructural capabilities. For example, more developed transport systems may make social distancing easier. Foreign direct investment (FDI) and trade openness are included to control for effects of travel and information linkages between countries, which might help in information sharing, or sharing of best practices in solving challenges posed by the pandemic. Trade openness may offer advantages in knowledge flows and sharing innovative approaches and dissemination of scientific information on pandemic response. Border closures and vaccine nationalism may exacerbate the pandemic response by reducing trade openness, even as they reduce the spread of COVID-19 variants. Democracy is included to control for differences between countries in political institutions, and both educational and healthcare spending is included to control for differences in human capital and health infrastructure. Finally, the proportion of a country’s population over the age of 65 is included, to control for differences in age distributions in Covid vulnerability. Having reviewed relevant literature, the methodology of the study is now discussed.

## 3. Methodology

Our empirical strategy proceeds as follows. First, descriptive analysis is performed and reported in Table 1. Bivariate analysis is then applied to obtain baseline zero-order correlations between our variables of interest, reported in Tables 2–5. Country-level cross-sectional regression models are used to test the direct and interactive associations of cultural norms and selected cultural values with pre-vaccine COVID-19 cases and deaths. Table 6 reports the direct associations of the Gelfand et al. tightness measure and Uz’s looseness measure. Table 7 reports their interactions with scientific capacity. Table 8 then reports additional interaction models testing whether selected cultural value dimensions, namely indulgence, individualism, and in-group collectivism, moderate the association between scientific capacity and COVID-19 cases and deaths. All models include the full set of covariates, with interaction models including the relevant interaction term.

**Table 1:** Descriptive statistics.

	count	mean	sd	min	max
Log COVID-19 cases per million (pre-vaccine)	154	8.148	2.131	1.792	10.870
Log COVID-19 deaths per million (pre-vaccine)	149	4.228	2.014	−2.526	7.329
Log COVID-19 tests per million (pre-vaccine)	142	11.178	1.623	6.360	14.407
Gelfand Tightness	56	0.054	0.441	−0.604	1.243
Uz Looseness	61	52.580	25.871	0.000	119.800
Hofstede Power Distance	68	58.912	21.438	11.000	100.000
Hofstede Individualism	68	44.147	24.650	6.000	91.000
Hofstede Masculinity	68	48.618	19.520	5.000	100.000
Hofstede Uncertainty Avoidance	68	66.971	23.224	8.000	100.000
Hofstede Long Term Orientation	88	45.721	24.400	0.000	100.000
Hofstede Indulgence	87	44.386	22.543	0.000	100.000
GLOBE Uncertainty Avoidance	56	4.666	0.592	3.158	5.610
GLOBE Future Orientation	56	5.472	0.526	2.952	6.203
GLOBE Power Distance	56	2.765	0.405	2.041	4.355
GLOBE Institutional Collectivism	56	4.741	0.511	3.826	5.654
GLOBE In-Group Collectivism	56	5.658	0.403	4.063	6.519
GLOBE Humane Orientation	56	5.380	0.371	3.394	6.087
GLOBE Performance Orientation	56	5.879	0.576	2.348	6.577
GLOBE Gender Egalitarianism	56	4.469	0.497	3.182	5.172
GLOBE Assertiveness	56	3.850	0.662	2.660	5.564
Log GDP per capita, 2015–2017 mean	158	8.603	1.488	5.397	11.414
Log(1 + scientific outputs per million), 2015–2017 mean	156	4.266	2.118	0.357	7.878
Fixed capital formation (% GDP), 2015–2017 mean	146	22.873	6.502	5.751	42.258
FDI net inflows (% GDP) 2015–2017 mean	151	4.163	6.723	−7.758	45.631
Trade openness (%GDP) 2015–2017 mean	158	42.168	29.776	0.307	205.038
Polity/democracy index, 2015–2017 mean	152	4.061	6.260	−10.000	10.000
Education spending (% GDP), 2015–2017 mean	107	4.427	1.360	1.340	7.764
Health expenditure (% GDP), 2015–2017 mean	150	6.649	2.652	2.140	17.853
Unemployment (% total labour force), 2015–2017 mean	160	7.669	5.952	0.141	27.334
Population aged 65+ (% total population), 2015–2017 mean	159	8.655	6.341	0.985	26.574

Note. Economic, scientific-capacity, demographic, and institutional covariates are measured as 2015–2017 country-level means unless otherwise stated. COVID-19 cases, deaths, and tests are logged pre-vaccine measures. Abbreviations are used in subsequent tables.

**Table 2:** Correlations between COVID-19 response variables and societal tightness values.

	Log COVID-19 cases per million (pre-vaccine)	Log COVID-19 deaths per million (pre-vaccine)	Log COVID-19 tests per million (pre-vaccine)	tightness	looseness
Log COVID-19 cases per million (pre-vaccine)	1.000				
Log COVID-19 deaths per million (pre-vaccine)	0.899^***^	1.000			
Log COVID-19 tests per million (pre-vaccine)	0.731^***^	0.519^***^	1.000		
tightness	−0.505^***^	−0.546^***^	−0.346^**^	1.000	
looseness	0.503^***^	0.509^***^	0.556^***^	−0.480^***^	1.000

*t* statistics in parentheses ^*^ *p* < 0.10, ^**^ *p* < 0.05, ^***^ *p* < 0.01.

**Table 3:** Correlations between COVID-19 response variables and Hofstede values.

	Log COVID-19 cases per million	Log COVID-19 deaths per million	Log COVID-19 tests per million	Hofstede Power Distance	Hofstede Individualism	Hofstede Masculinity	Hofstede Uncertainty Avoidance	Hofstede Long Term Orientation	Hofstede Indulgence
Log COVID-19 cases per million	1.000								
Log COVID-19 deaths per million	0.925^***^	1.000							
Log COVID-19 tests per million	0.600^***^	0.446^***^	1.000						
Hofstede Power Distance	−0.092	−0.108	−0.408^***^	1.000					
Hofstede Individualism	0.317^**^	0.323^**^	0.614^***^	−0.651^***^	1.000				
Hofstede Masculinity	−0.022	0.023	−0.203	0.143	0.044	1.000			
Hofstede Uncertainty Avoidance	0.304^**^	0.403^***^	−0.103	0.258^*^	−0.246^*^	0.053	1.000		
Hofstede Long Term Orientation	0.077	−0.033	0.207	0.010	0.165	−0.001	0.019	1.000	
Hofstede Indulgence	0.040	0.101	0.031	−0.288^**^	0.135	0.099	−0.120	−0.528^***^	1.000

*t* statistics in parentheses. ^*^ *p* < 0.10, ^**^ *p* < 0.05, ^***^ *p* < 0.01. COVID-19 cases, deaths, and tests are pre-vaccine.

**Table 4:** Correlations between COVID-19 response variables and GLOBE cultural values.

	Log COVID-19 cases per million (pre-vaccine)	Log COVID-19 deaths per million (pre-vaccine)	Log COVID-19 tests per million (pre-vaccine)	Uncertainty Avoidance	Future Orientation	Power Distance	Institutional Collectivism	In-Group Collectivism	Humane Orientation	Performance Orientation	Gender Egalitarianism	Assertiveness
Log COVID-19 cases per million	1.000											
Log COVID-19 deaths per million	0.888^***^	1.000										
Log COVID-19 tests per million	0.576^***^	0.368^***^	1.000									
Uncertainty Avoidance	−0.361^***^	−0.250^*^	−0.603^***^	1.000								
Future Orientation	−0.275^**^	−0.247^*^	−0.511^***^	0.652^***^	1.000							
Power Distance	−0.141	−0.205	0.002	−0.011	−0.373^***^	1.000						
Institutional Collectivism	0.026	0.117	−0.358^***^	0.451^***^	0.522^***^	−0.386^***^	1.000					
In-Group Collectivism	−0.042	0.026	−0.223	0.367^***^	0.651^***^	−0.407^***^	0.392^***^	1.000				
Humane Orientation	0.045	−0.000	0.090	0.082	0.411^***^	−0.614^***^	0.095	0.261^*^	1.000			
Performance Orientation	−0.069	−0.005	−0.207	0.329^**^	0.730^***^	−0.584^***^	0.458^***^	0.714^***^	0.593^***^	1.000		
Gender Egalitarianism	0.342^**^	0.414^***^	0.331^**^	−0.445^***^	−0.145	−0.514^***^	0.040	0.227^*^	0.256^*^	0.260^*^	1.000	
Assertiveness	−0.317^**^	−0.310^**^	−0.158	0.172	−0.006	0.308^**^	−0.212	−0.081	−0.126	−0.062	−0.297^**^	1.000

*t* statistics in parentheses. ^*^ *p* < 0.10, ^**^ *p* < 0.05, ^***^ *p* < 0.01.

**Table 5:** Correlations between pre-vaccine COVID-19 response variables and economic variables.

	Log COVID-19 cases per million	Log COVID-19 deaths per million	Log COVID-19 tests per million	Log GDP per capita	Log(1 + mean scientific outputs per million)	Fixed Capital Formation	FDI	Trade Openness	Polity/ Democracy	Education Spending	Health Expenditure	Unemployment	Population aged 65+
Log COVID-19 cases per million	1.000												
Log COVID-19 deaths per million	0.946^***^	1.000											
Log COVID-19 tests per million	0.773^***^	0.677^***^	1.000										
Log GDP per capita	0.646^***^	0.614^***^	0.854^***^	1.000									
Log(1 + mean scientific outputs per million)	0.628^***^	0.580^***^	0.836^***^	0.920^***^	1.000								
Fixed Capital Formation	−0.152	−0.176^*^	−0.127	−0.112	−0.184^*^	1.000							
FDI	0.106	0.102	0.105	0.141	0.095	0.223^**^	1.000						
Trade Openness	0.277^***^	0.231^**^	0.423^***^	0.445^***^	0.456^***^	0.044	0.435^***^	1.000					
Polity/Democracy	0.307^***^	0.353^***^	0.272^***^	0.442^***^	0.412^***^	−0.177^*^	0.116	0.128	1.000				
Education spending	0.164	0.155	0.259^**^	0.339^***^	0.354^***^	−0.140	0.018	0.156	0.376^***^	1.000			
Health expenditure	0.320^***^	0.356^***^	0.341^***^	0.480^***^	0.459^***^	−0.295^***^	0.101	0.146	0.385^***^	0.457^***^	1.000		
Unemployment	0.232^**^	0.256^**^	0.085	0.050	0.122	−0.111	0.156	−0.037	0.177^*^	0.227^**^	0.256^**^	1.000	
Population aged 65+	0.573^***^	0.528^***^	0.746^***^	0.836^***^	0.851^***^	−0.175^*^	0.084	0.394^***^	0.523^***^	0.285^***^	0.542^***^	0.090	1.000

*t* statistics in parentheses. ^*^ *p* < 0.10, ^**^ *p* < 0.05, ^***^ *p* < 0.01. Note. See Table 1 for full variable definitions and units. COVID-19 cases, deaths, and tests are logged pre-vaccine measures. Economic, scientific-capacity, demographic, and institutional covariates are measured as 2015–2017 country-level means.

**Table 6:** Direct associations of scientific capacity and tightness/looseness.

	(1)	(2)	(3)	(4)
	Cases-Looseness	Deaths-Looseness	Cases-Tightness	Deaths-Tightness
looseness	0.011	0.014		
	(0.008)	(0.009)		
Scientific capacity (std.)	0.792	0.660	0.482	0.346
	(0.724)	(0.697)	(0.458)	(0.539)
GDP per capita (std.)	−0.215	0.135	0.100	0.296
	(0.643)	(0.639)	(0.407)	(0.444)
Fixed capital formation (std.)	−0.235	−0.332	−0.410^*^	−0.377
	(0.300)	(0.305)	(0.223)	(0.284)
FDI (std.)	0.061	0.090	0.025	0.006
	(0.095)	(0.104)	(0.094)	(0.105)
Trade openness (std.)	−0.112	−0.205	−0.114	−0.160
	(0.264)	(0.252)	(0.208)	(0.207)
Polity/democracy (std.)	0.226	0.225	0.352	0.311
	(0.338)	(0.301)	(0.217)	(0.208)
Education spending % GDP (std.)	−0.033	0.001	−0.178	−0.260
	(0.174)	(0.207)	(0.167)	(0.213)
Health expenditure (std.)	−0.270	−0.404	−0.025	0.170
	(0.251)	(0.258)	(0.184)	(0.209)
Unemployment (std.)	0.135	0.129	0.130	0.113
	(0.137)	(0.153)	(0.122)	(0.141)
Population aged 65+ (std.)	−0.057	−0.237	−0.268	−0.407^*^
	(0.235)	(0.235)	(0.172)	(0.233)
tightness			−0.653^**^	−1.057^***^
			(0.277)	(0.338)
Constant	−0.537	−0.552	0.046	0.100
	(0.552)	(0.547)	(0.208)	(0.213)
N	42	42	40	40
R-squared	0.494	0.523	0.639	0.652

* *p* < 0.10, ^**^ *p* < 0.05, ^***^ *p* < 0.01. See Table 1 for full variable definitions and units. Standard errors in parentheses. Country-level cross-section only. Outcomes are standardised logged COVID-19 outcomes. Predictors are standardised except looseness; tightness is already standardised in the source data. COVID-19 cases, deaths, and tests are logged pre-vaccine measures. Economic, scientific-capacity, demographic, and institutional covariates are measured as 2015–2017 country-level means.

**Table 7:** Interactions of scientific capacity with tightness/looseness.

	(1)	(2)	(3)	(4)
	Cases-Looseness	Deaths-Looseness	Cases-Tightness	Deaths-Tightness
looseness	0.016	0.019^*^		
	(0.010)	(0.010)		
Scientific capacity (std.)	1.024	0.911	0.543	0.463
	(0.748)	(0.774)	(0.460)	(0.552)
looseness # Scientific capacity (std.)	−0.005	−0.006		
	(0.008)	(0.008)		
GDP per capita (std.)	−0.206	0.145	−0.032	0.044
	(0.629)	(0.620)	(0.389)	(0.419)
Fixed capital formation (std.)	−0.210	−0.305	−0.415^*^	−0.387
	(0.292)	(0.297)	(0.237)	(0.305)
FDI (std.)	0.063	0.092	0.076	0.104
	(0.100)	(0.109)	(0.099)	(0.099)
Trade openness (std.)	−0.119	−0.212	−0.125	−0.181
	(0.265)	(0.254)	(0.203)	(0.187)
Polity/democracy (std.)	0.224	0.223	0.388^*^	0.379^*^
	(0.322)	(0.283)	(0.224)	(0.206)
Education spending % GDP (std.)	−0.041	−0.007	−0.151	−0.209
	(0.168)	(0.203)	(0.160)	(0.191)
Health expenditure (std.)	−0.256	−0.389	−0.079	0.066
	(0.258)	(0.265)	(0.198)	(0.229)
Unemployment (std.)	0.133	0.127	0.130	0.114
	(0.142)	(0.159)	(0.119)	(0.138)
Population aged 65+ (std.)	−0.053	−0.233	−0.230	−0.336
	(0.238)	(0.236)	(0.173)	(0.225)
tightness			−0.784^**^	−1.309^***^
			(0.291)	(0.355)
tightness # Scientific capacity (std.)			0.388	0.740
			(0.446)	(0.567)
Constant	−0.693	−0.720	0.094	0.191
	(0.597)	(0.589)	(0.206)	(0.208)
N	42	42	40	40
R-squared	0.504	0.533	0.649	0.676

Standard errors in parentheses. ^*^ *p* < 0.10, ^**^ *p* < 0.05, ^***^ *p* < 0.01. See Table 1 for full variable definitions and units. COVID-19 cases, deaths, and tests are logged pre-vaccine measures. Economic, scientific-capacity, demographic, and institutional covariates are measured as 2015–2017 country-level means. A positive interaction coefficient indicates that the association between scientific capacity and the outcome becomes more positive as the moderator increases; a negative interaction coefficient indicates that this association becomes less positive or more negative as the moderator increases.

**Table 8:** Interactions of scientific capacity with indulgence, individualism, and in-group collectivism.

	(1)	(2)	(3)	(4)	(5)	(6)
	Cases-Indulgence	Deaths-Indulgence	Cases-Individualism	Deaths-Individualism	Cases-In-group collectivism	Deaths-In-group collectivism
Hofstede indulgence (std.)	0.021	0.041				
	(0.150)	(0.194)				
Scientific capacity (std.)	0.232	−0.067	0.377	0.214	−0.005	−0.068
	(0.330)	(0.395)	(0.478)	(0.559)	(0.396)	(0.454)
Hofstede indulgence (std.) # Scientific capacity (std.)	−0.294^*^	−0.235				
	(0.156)	(0.174)				
GDP per capita (std.)	0.258	0.558	0.243	0.368	0.091	0.164
	(0.333)	(0.364)	(0.524)	(0.662)	(0.495)	(0.502)
Fixed capital formation (std.)	−0.156	−0.243	−0.352^*^	−0.341	−0.251	−0.284
	(0.143)	(0.147)	(0.189)	(0.208)	(0.175)	(0.206)
FDI (std.)	0.269^**^	0.251	0.040	0.066	0.045	0.045
	(0.132)	(0.151)	(0.105)	(0.126)	(0.149)	(0.152)
Trade openness (std.)	−0.179	−0.173	0.053	0.005	0.198	0.184
	(0.227)	(0.249)	(0.167)	(0.195)	(0.250)	(0.269)
Polity/democracy (std.)	−0.041	−0.013	0.398	0.352	−0.141	−0.088
	(0.217)	(0.203)	(0.406)	(0.435)	(0.181)	(0.226)
Education spending (std.)	0.109	0.103	−0.052	−0.116	0.109	0.012
	(0.158)	(0.177)	(0.160)	(0.178)	(0.181)	(0.206)
Health expenditure (std.)	−0.067	−0.142	−0.044	0.060	−0.076	0.066
	(0.201)	(0.252)	(0.210)	(0.283)	(0.247)	(0.287)
Unemployment (std.)	0.282^**^	0.375^***^	0.318^*^	0.390^*^	0.490^**^	0.544^*^
	(0.108)	(0.128)	(0.179)	(0.203)	(0.233)	(0.272)
Population aged 65+ (std.)	0.108	0.090	−0.127	−0.211	0.124	−0.009
	(0.264)	(0.285)	(0.295)	(0.344)	(0.253)	(0.293)
Hofstede individualism (std.)			−0.295^**^	−0.304^*^		
			(0.134)	(0.168)		
Hofstede individualism (std.) # Scientific capacity (std.)			0.118	0.169		
			(0.174)	(0.201)		
GLOBE in-group collectivism (std.)					0.084	0.095
					(0.216)	(0.240)
GLOBE in-group collectivism (std.) # Scientific capacity (std.)					−0.166	−0.170
					(0.173)	(0.203)
Constant	0.115	0.189	−0.158	−0.016	0.428	0.529^*^
	(0.176)	(0.185)	(0.298)	(0.327)	(0.256)	(0.285)
N	61	60	48	48	36	36
R-squared	0.499	0.471	0.447	0.357	0.447	0.366

See Table 1 for full variable definitions and units. Standard errors in parentheses. ^*^ *p* < 0.10, ^**^ *p* < 0.05, ^***^ *p* < 0.01 Note. Individualism and indulgence are Hofstede cultural value dimensions. In-group collectivism is from the GLOBE values measure. Scientific capacity is measured as the log of one plus mean scientific outputs per million people, 2015–2017. COVID-19 cases, deaths, and tests are logged pre-vaccine measures. Economic, scientific-capacity, demographic, and institutional covariates are measured as 2015–2017 country-level means.

### 3.1 Data and data collection

Scientific capacity is measured using a country’s scientific and technical journal publications, expressed per million people and logged after adding one. This measure is used as a country-level proxy for the stock and recent flow of codified scientific knowledge available for problem-solving. It is theoretically appropriate because the paper tests whether scientific knowledge capacity is associated with lower COVID-19 cases and deaths. In Romer’s framework, codified ideas are non-rival [55]. Once produced, they can be reused, adapted, and disseminated at low marginal cost. Oral rehydration therapy illustrates this logic, as knowledge of a simple formula combining water, sugar, and salts can be reproduced widely to prevent deaths from diarrhoeal disease. Applied to COVID-19, the same logic suggests that countries with greater scientific capacity should be better able to access, interpret, validate, and adapt knowledge about transmission, testing, treatment, public-health protocols, and behavioural interventions.

At the same time, this measure also allows the analysis to test Jones’s [56] qualification of simple knowledge-accumulation models, whereby the realised effects of scientific knowledge may be weakened by duplication, congestion, and absorptive limits. Scientific output per million is therefore an appropriate aggregate proxy for scientific capacity, while the empirical analysis tests whether this capacity was actually converted into lower pre-vaccine COVID-19 cases and deaths. The measure is also consistent with recombinant innovation arguments associated with Weitzman [9], because a larger stock of scientific output should increase the potential for recombination, adaptation, and problem-solving under crisis conditions.

#### Gelfand et al.’s tightness scale.

Gelfand et al. (2011) validate a cultural tightness scale across 33 countries, developing a scale to measure overall strength of social norms and tolerance of deviance. This scale is validated further by Eriksson et al. [64] in a sample of 57 countries and used in Gelfand et al. (2021). These scale items comprise six-item Likert scale measures assessing “the degree to which social norms are pervasive, clearly-defined and reliably imposed within nations” [3]. In initially developing this index Gelfand et al. (2011) adapted measures of behavioral constraint and situational appropriateness [65]. See Gelfand et al. (2011), Eriksson et al. [64], and Gelfand et al. (2021) for detailed information on scale construction and validity, and for access to the publicly available data.

This scale is used, referred to here as ‘tightness’, to differentiate it from our discussions of the standard deviation (SD)-based cultural tightness/looseness (CTL) index developed by Uz (2015), which is termed ‘looseness’ as higher values represent higher standard deviations of norms. Tightness is used in hypothesis testing, as it theoretically represents tightness as a cultural norm, and looseness, a measure of cultural homogeneity of a country, is tested to generate further insights.

#### Uz’s SD looseness scale.

According to Uz [36], cultural tightness reflects homogeneity in values, norms, and behaviours captured by standard deviation (SD) in survey responses. Uz develops an index of CTL norms for 68 countries. She argues that acquiescent response style bias due to excessively positive responses to questions, and extreme response style bias caused by the use of extreme ends of item response scales should not necessarily be addressed by removing differences in SD (Uz, 2015:324), in that these types of bias concern extremity in individual responses whereas standard deviation captures variation across individuals. More extreme responses by multiple respondents can therefore also result in smaller SD, and such effects on SD can therefore be *a priori* indeterminate.

Using data from the year 2000 sampling wave of the European Values Study Group and World Survey [66], Uz (2015) develops scale items for general (g-factor) and specific (s-factor) domain measures of statistical dispersion of CTL, as well as a combination measure. The underlying data is derived from 101 172 responses over 70 countries, resulting in a final data set for 68 countries. The domain specific index was derived from EWVS questions on morality, and the domain general index from all available data. The final index used factor analysis of groups of domains to extract the CTL index. This final CTL index is used here. See Uz (2015) for further information about the derivation of the CTL index and its validation and for access to this publicly available data. Further testing using Uz’s looseness index provides comparative insight into whether Gelfand’s cultural tightness norms are similar in their associations to homogenous cultural norms.

#### Hofstede’s comprehensive values scale.

Hofstede’s [67] initial development of scale measures used an inductive analysis of over 100000 data responses from the company IBM across 50 countries. Further analysis using ecological factor analysis ultimately led to the development of six dimensions of societal culture. The Hofstede index has items on a scale that runs largely between 0 and 100 [63]. Technical details of his methodological approach and data used here is obtained from Hofstede’s publicly available data site [63]. Two indicators are tested, namely indulgence versus restraint, and individualism, in that Hofstede (2020) specifically hypothesized about how these might contribute to COVID-19 outcomes in the early stages of the pandemic. Correlations are reported between all items of all four norms/values frameworks in Tables 2–5, even though our further analysis is restricted to three items that are included for their theoretical overlap with tightness norms.

#### GLOBE’s comprehensive values scale.

GLOBE cultural values items comprise data drawn from 17 000 respondents across 951 organisations, 62 countries, and all major world regions [8]. Their development follows a multistage process including qualitative evaluations in the form of item review, back translation, Q-sorting, and quantitative evaluation techniques including correlation analysis, multilevel confirmatory factor analysis, as well as a range of other scale evaluation techniques (for more detailed information, see Hanges and Dickson [68]). GLOBE cultural values were tested, rather than practices, because contingencies and many different influences can shape practices, more so than values, and our focus here is delimited to norms and values.

According to Hofstede [35], his analysis shows “that in spite of a very different approach, the massive body of GLOBE data still reflected the structure of the original Hofstede model”, except humane and performance orientations. GLOBE researchers suggest that their analysis is statistically rigorous, and that their dimensions address confounds in Hofstede’s work, to advance it theoretically and empirically [69]. A notable difference in the GLOBE approach concerns their split of the individualism/collectivism values item into in-group and institutional collectivism. Their in-group collectivism item is tested here. Considering an extensive body of work that has used Hofstede’s dimensions, and the fact that both Hofstede and GLOBE frameworks have been extensively validated using correlation analysis with large numbers of contextual variables, a collectivism/individualism measure is used from both to derive complementary insights because they disagree in certain theoretical respects. These differences allow us to derive nuanced insights in testing.

#### Coronavirus response data.

Our COVID-19 response data comes from the data aggregation site Worldometer [70]. In this respect, the analysis follows a growing body of other studies that use the same source [71–74]. Three measures are included in testing, namely COVID-19 cases, deaths, and tests conducted per million of a country’s population. Natural logs of these variables are used in testing. Data was collected on the 19^th^ of December 2020. This date was chosen as it was prior to announcements of vaccine approvals in some countries of the world which might have resulted in a structural break in compliance with NPIs, or country differences due to inequality in vaccine access. As such, this period covers the pre-vaccine period of the pandemic. It also avoids confounds associated with differences between countries that celebrate a December holiday period and those that do not.

### 3.2 Empirical approach

The following specifications are tested using country-level cross-sectional regression models. First, a baseline covariate model is estimated, drawing on the literature discussed above.


$$\begin{array}{cc} COVID-\text{1}\text{9}\;{outcome}_{i}= & \;\beta_{\text{0}}+\beta_{\text{1}}{GDPpc}_{i}+\beta_{\text{2}}{investment}_{i}+\beta_{\text{3}}{FDI}_{i}+\beta_{\text{4}}{tradeopen}_{i}+\beta_{\text{5}}{democracy}_{i}\\ & +\beta_{\text{6}}{edspend}_{i}+\beta_{\text{7}}{healthexp}_{i}+\beta_{\text{8}}{unemp}_{i}+\beta_{\text{9}}{ageover\text{6}\text{5}}_{i}+\beta_{\text{1}\text{0}}{tests}_{i}+\varepsilon_{i} \end{array}$$ (1)

The covariates in equation (1) are represented by *Z*_i_ in the following models. Scientific capacity is denoted by *S*_i_. COVID-19 outcomes refer in each instance to either cases or deaths, which are tested and reported separately. The economic, scientific-capacity, demographic, and institutional covariates are measured as 2015–2017 country-level means to capture pre-pandemic structural conditions while reducing sensitivity to single-year fluctuations. Using pre-COVID-19 averages also helps avoid incorporating pandemic-period changes that could be endogenous to COVID-19 cases, deaths, testing, and policy responses.

Direct associations between tightness or looseness and COVID-19 outcomes are first estimated as follows:


$$\begin{array}{c} COVID-\text{1}\text{9}\;{outcome}_{i}=\beta_{\text{0}}+\beta_{\text{1}}T_{i}+\beta_{\text{2}}S_{i}+{\gamma Z}_{i}+\varepsilon_{i} \end{array}$$ (2)

$$\begin{array}{c} COVID-\text{1}\text{9}\;{outcome}_{i}=\beta_{\text{0}}+\beta_{\text{1}}L_{i}+\beta_{\text{2}}S_{i}+{\gamma Z}_{i}+\varepsilon_{i} \end{array}$$ (3)

where *T*_*i*_ denotes Gelfand et al.’s tightness measure and *L*_*i*_ denotes Uz’s looseness measure. Interaction models are then estimated to test whether the associations between tightness or looseness and COVID-19 outcomes vary according to scientific capacity:


$$\begin{array}{c} COVID-\text{1}\text{9}\;{outcome}_{i}=\beta_{\text{0}}+\beta_{\text{1}}T_{i}+\beta_{\text{2}}S_{i}+\beta_{\text{3}}(T_{i}\times S_{i})+{\gamma Z}_{i}+\varepsilon_{i} \end{array}$$ (4)

$$\begin{array}{c} COVID-\text{1}\text{9}\;{outcome}_{i}=\beta_{\text{0}}+\beta_{\text{1}}L_{i}+\beta_{\text{2}}S_{i}+\beta_{\text{3}}(L_{i}\times S_{i})+{\gamma Z}_{i}+\varepsilon_{i} \end{array}$$ (5)

$$\begin{array}{c} COVID-\text{1}\text{9}\;{outcome}_{i}=\beta_{\text{0}}+\beta_{\text{1}}V_{i}+\beta_{\text{2}}S_{i}+\beta_{\text{3}}(V_{i}\times S_{i})+{\gamma Z}_{i}+\varepsilon_{i} \end{array}$$ (6)

where *V*_*i*_ denotes the selected cultural value dimension for country *i*, replaced in turn by Hofstede indulgence, Hofstede individualism, and GLOBE in-group collectivism. These models test whether these cultural value dimensions moderate the association between scientific capacity and COVID-19 cases and deaths. In these models, *β*_*0*_ is the constant term and *ε*_i_ is the residual error component for country *i*. The focal explanatory variables are defined as follows:

*T*_*i*_ = Gelfand et al.’s tightness measure for country *i*.
*L*_*i*_ = Uz’s looseness measure for country *i*.
S_i_ = Scientific capacity for country *i*, measured as the natural log of one plus mean scientific outputs per million people, 2015–2017.
*V*_*i*_ = The selected cultural value dimension used in the additional interaction models. In these models, *V*_*i*_ is replaced in turn by Hofstede individualism, Hofstede indulgence, and GLOBE in-group collectivism.

The vector *Z*_*i*_ includes the following covariates:

*GDP =*  Natural log of GDP per capita measured at 2011 US purchasing power parity prices.
*investment*   =  Fixed domestic capital formation as a share of GDP.
*FDI*  =  Net inflows of foreign direct investment as a share of GDP.
*trade openness*  =  Trade, measured as exports plus imports as a share of GDP.
*democracy  =*  Democracy index measure.
*edspend*  =  Education expenditure as a share of GDP.
*healthexp  =*  Healthcare expenditure as a share of GDP.
*unemployment  =*  Unemployment as a share of the labour force.
*ageover65  =*  Population aged 65 and over as a share of the population.
*tests*  =  Log COVID-19 tests per million during the pre-vaccine period.

Per capita GDP data is obtained from the Penn World Tables [75], investment, FDI, and trade openness data from the United Nations National Accounts Main Aggregates Database (NAMAD)- United Nations [76], and the educational expenditure, health expenditure, unemployment, and age data from the World Bank Development Indicators- World Bank [77]. The democracy indicator is obtained from the Center for Systemic Peace Polity database [78].

As discussed, Table 6 reports direct associations of scientific capacity and the two tightness/looseness indicators with COVID-19 cases and deaths. Table 7 extends these models by adding interaction terms between scientific capacity and tightness/looseness. Although correlations are reported for the full set of Hofstede and GLOBE cultural value dimensions, the subsequent regression-based interaction tests focus only on the theoretically selected dimensions most closely related to the tightness mechanism: Hofstede indulgence, Hofstede individualism, and GLOBE in-group collectivism. These tests are reported in Table 8.

Overall, the empirical approach combines established theoretical frameworks and scales with publicly available cross-national data, supporting transparency, replication, and comparison with related research.

## 4. Results

The bivariate correlations provide initial descriptive support for the central expectation that cultural tightness and looseness are associated with different pandemic outcomes. Tightness is negatively correlated with pre-vaccine COVID-19 cases and deaths, whereas looseness is positively correlated with both outcomes. This pattern is notable because it suggests that tighter norm enforcement may have been associated with lower pandemic burden during the pre-vaccination phase, while looser cultural environments may have been associated with higher cases and deaths. Looseness is also positively correlated with testing, whereas tightness is negatively correlated with testing, indicating that observed case differences may partly reflect differences in testing intensity, but not only this, since the same broad pattern is also observed for deaths.

The correlations with broader cultural value dimensions reveal several interesting and less straightforward patterns. Hofstede individualism is positively correlated with cases, deaths, and testing, consistent with the possibility that more individualistic societies may have faced greater difficulty sustaining collective non-pharmaceutical interventions. Hofstede uncertainty avoidance is also positively correlated with cases and deaths, which is less expected if uncertainty avoidance is assumed to support caution and compliance. By contrast, power distance is strongly negatively correlated with testing but is not significantly associated with cases or deaths, suggesting that hierarchical cultural structures may relate more clearly to measurement and reporting capacity than to pandemic outcomes directly.

The GLOBE values correlations add further nuance. GLOBE uncertainty avoidance and future orientation are negatively correlated with cases, deaths, and testing, suggesting that societies valuing planning, rule-following, and future-oriented behaviour may have experienced lower pre-vaccine pandemic burden. However, one of the most unexpected findings is that gender egalitarianism is positively correlated with cases, deaths, and testing. This may reflect its association with broader social, institutional, or developmental characteristics rather than a direct cultural effect. Assertiveness, by contrast, is negatively correlated with cases and deaths, which is also somewhat unexpected and suggests that some cultural dimensions may not map neatly onto the tightness–looseness mechanism.

The economic and scientific-capacity correlations also require careful interpretation. GDP per capita and scientific output are strongly positively correlated with testing, but also with cases and deaths. This does not necessarily imply that wealth or scientific capacity worsened pandemic outcomes. Rather, these correlations likely reflect the fact that richer and more scientifically developed countries had greater testing capacity, older populations, more international connectivity, and more complete detection and reporting. The strong positive correlations between scientific output, GDP per capita, testing, and population ageing therefore reinforce the need for multivariate models that separate detection capacity and structural exposure from substantive pandemic response effects.

Overall, the correlation results suggest that the tightness–looseness pattern is descriptively clear, but the wider cultural values results are more mixed and theoretically interesting. Some values appear to approximate the expected tightness mechanism, especially GLOBE uncertainty avoidance and future orientation, whereas others, such as Hofstede uncertainty avoidance, gender egalitarianism, and assertiveness, point to more complex or unexpected relationships. These findings justify the subsequent interaction tests with scientific capacity, since the bivariate associations suggest that cultural response mechanisms and scientific capability may not operate independently in shaping pre-vaccine pandemic outcomes.

Regression results show associations of tightness remain robust. Scientific capacity does not show a direct protective effect. Most interactions are not significant, and do not support a clearly “indirect/synergistic/antagonistic” interpretation. Accordingly, whereas direct cultural tightness effects are robust, scientific-capacity effects and interaction effects are weak or non-robust. Scientific capacity is positively correlated with reported COVID-19 cases and deaths in Table 5 but is not significantly associated with cases or deaths in the multivariate models in Tables 6–8. Tightness cannot be abstracted away in a regression without introducing omitted variable bias. Hypothesis A, that scientific capacity is directly and negatively associated with COVID-19 cases, and Hypothesis B with deaths, are not supported. Results of hypothesis testing are reported in Table 9. Gelfand tightness has significant direct effects, being negatively and significantly associated with both cases and deaths in Tables 6 and 7. Hypotheses C and D are therefore supported. Uz looseness has the expected positive direction, but it is mostly not significant, except weakly for deaths in Table 7. In terms of interaction effects, Table 7 shows no significant interaction between scientific capacity and tightness/looseness. Table 8 shows only one weak interaction, indulgence × scientific capacity for cases. Hypotheses E and F are not supported, there being no robust evidence that cultural tightness significantly weakens any scientific-capacity association with cases, or deaths, respectively.

**Table 9:** Results of hypothesis testing of associations with COVID-19 cases and deaths.

Hypotheses	Hypothesized Associations	Outcome
Hypothesis A	Scientific capacity is directly negatively associated with cases	Not Supported
Hypothesis B	Scientific capacity is directly negatively associated with deaths	Not Supported
Hypothesis C	Tightness* is directly negatively associated with cases	Supported
Hypothesis D	Tightness* is directly negatively associated with deaths.	Supported
Hypothesis E	Cultural tightness weakens the protective association between scientific capacity and cases.	Not Supported
Hypothesis F	Cultural tightness weakens the protective association between scientific capacity and deaths.	Not Supported

*Tightness refers to Gelfand tightness. Further analysis using Uz looseness shows coefficients in the expected direction for Hypotheses C and D, but looseness is only weakly significant for deaths. Additional values-based interaction tests find only one weak interaction, a negative interaction between Hofstede indulgence and scientific capacity for cases.

Further tests reported in Table 8 find Hofstede individualism to be directly and negatively associated with cases and weakly with deaths, but its interaction with scientific capacity is not significant, whereas indulgence has no direct effect, but weakly moderates cases, the sign of the coefficient being negative. The indulgence interaction suggests weak evidence that scientific capacity was more beneficial in indulgent societies. At higher levels of indulgence, the association between scientific capacity and COVID-19 cases becomes less positive and may become slightly negative. This suggests that, where societies allow greater individual discretion and quality-of-life orientation, scientific knowledge may have translated more effectively into behavioural or policy responses. However, because the interaction is only weakly significant and the corresponding death interaction is not significant, this should be interpreted cautiously.

Table 8 suggests in-group collectivism also has non-significant associations. Scientific capacity does not seem to work synergistically in a fully significant way with any of norm or culture indicators. There is therefore no significant evidence to suggest that tightness and science work orthogonally or interactively to reduce deaths. Tightness has robust direct associations, but any evidence of moderation is limited.

## 5. Discussion

This study examined whether scientific capacity and cultural tightness were associated with pre-vaccine COVID-19 cases and deaths, and whether cultural tightness moderated the relationship between scientific capacity and pandemic outcomes. The results provide a cautious but theoretically important account of this relationship, specifically extending literature concerned with evaluating the effectiveness of scientific research [22–30], particularly regarding the COVID-19 pandemic [31,32], and its management protocols [38]. Accordingly, scientific capacity was expected to reduce cases and deaths by enabling countries to access, interpret, validate, and apply emerging scientific knowledge about transmission, testing, treatment, behavioural interventions, and public-health protocols. This expectation is consistent with endogenous growth and recombinant innovation perspectives, which suggest that scientific knowledge enlarges the feasible set of interventions, improves problem-solving capacity, and enables ideas to be reused and recombined across contexts [9–11,54–56]. However, the findings do not support a robust direct protective association between scientific capacity and either COVID-19 cases or deaths once cultural tightness/looseness and structural covariates are included.

This should not be interpreted as evidence that scientific capacity was irrelevant. Rather, it suggests that scientific capacity is not automatically converted into population-level protection. Scientific capacity may enable countries to generate and absorb knowledge, participate in cross-national scientific networks, access global research, and adapt scientific insights to local conditions [39–43,60].

Scientific capacity is also a central component of national innovative capability and national innovation systems [44–53]. However, during the pre-vaccine phase of COVID-19, scientific knowledge had to be translated into coordinated behaviour, including isolation, distancing, mask wearing, testing, treatment-seeking, and compliance with rapidly changing public-health guidance [57–59]. Scientific capacity may therefore have enlarged the feasible set of possible responses, but the results suggest that this was insufficient, by itself, to reduce cases or deaths across countries. The effectiveness of scientific capacity might therefore have depended not only on the availability of knowledge, but also on communication, trust, institutional implementation, public compliance, and behavioural uptake.

The strongest and most consistent finding is the association between Gelfand cultural tightness and lower COVID-19 cases and deaths. This is consistent with previous findings that tighter societies experienced lower COVID-19 cases and deaths [1,2], and with the wider tightness–looseness argument that societies with stronger norms and lower tolerance of deviance may respond more effectively to collective threat [3,5–7]. Cultural tightness refers to the strength of social norms and tolerance of deviant behaviour [3], and Gelfand’s early argument that culturally loose societies may need to “tighten up” during COVID-19 provides a direct theoretical link between tightness and pandemic response [4]. The present findings support the direct version of this argument, that countries with stronger norms and lower tolerance of deviance appear to have been better able to limit pre-vaccine COVID-19 outcomes. This interpretation is plausible because, before vaccination, reducing transmission depended heavily on the effective implementation of non-pharmaceutical interventions. Where pandemic response required collective restraint and behavioural conformity, tighter cultures may have had an advantage because public-health rules were more likely to be understood, followed, and socially reinforced.

The interaction results are weaker. The models do not provide robust evidence that cultural tightness systematically moderates the association between scientific capacity and COVID-19 outcomes. This weakens the stronger theoretical expectation that tightness and scientific capacity operate antagonistically in their relationship with pandemic outcomes. The logic behind that expectation remains theoretically plausible, that tightness may support collective compliance while also constraining behavioural variation, dissent, experimentation, and creativity, all of which may matter for scientific discovery and innovation [8–21]. However, the empirical results suggest a simpler interpretation. Cultural tightness has a robust direct protective association with pre-vaccine COVID-19 outcomes, whereas scientific capacity does not show a robust direct or conditional protective association in these models. Hypotheses A and B, predicting direct protective associations of scientific capacity with cases and deaths, are therefore not supported. Hypotheses C and D, predicting direct negative associations of tightness with cases and deaths, are supported. Hypotheses E and F, predicting that tightness weakens the protective association between scientific capacity and cases and deaths, are not supported.

The additional cultural values models refine this interpretation. Hofstede individualism is negatively associated with cases and weakly negatively associated with deaths in the multivariate models, although its interaction with scientific capacity is not significant. This is theoretically interesting because individualism is often treated as potentially problematic for collective compliance, yet Hofstede suggested early in the pandemic that individualistic societies may also respond quickly to changing external conditions [63]. Once structural covariates are included, individualism may therefore capture adaptive responsiveness, individual discretion, or decentralised behavioural adjustment, rather than simple non-compliance. This contrasts with the bivariate correlations, where individualism is positively correlated with cases, deaths, and testing, and underlines the importance of distinguishing zero-order associations from multivariate results. Indulgence provides only weak evidence of moderation for cases, suggesting that scientific capacity may have been less positively associated with cases in more indulgent societies. However, this result is only weakly significant and does not extend to deaths, so it should be interpreted cautiously. GLOBE in-group collectivism does not provide robust explanatory evidence in these models, despite its theoretical relevance to conformity, group loyalty, and collective action [8,35,60,68,69].

The stronger performance of the Gelfand tightness measure relative to Uz’s looseness measure is theoretically important. Uz’s measure captures cultural homogeneity through dispersion in values, norms, and behaviours [36], whereas Gelfand’s measure more directly captures the strength, clarity, and reliable enforcement of social norms [3,64]. The results therefore suggest that pandemic outcomes were more strongly associated with norm strength and enforceability than with cultural homogeneity alone. This distinction matters because societies may share norms without those norms being strongly enforced, and they may be culturally homogeneous without being behaviourally restrictive. During the pre-vaccine phase, the protective mechanism appears to have been less about people thinking similarly and more about whether behavioural expectations were clear, binding, and socially reinforced. In this sense, the findings suggest that tightness is not reducible to homogeneity, but may operate specifically through norm clarity, social sanction, and reduced behavioural variance under threat.

At the same time, cultural tightness should not be treated as a general policy ideal. The results suggest that tightness may have been protective under the specific conditions of the pre-vaccine pandemic phase, when immediate response depended heavily on behavioural compliance. They do not imply that tighter societies are generally superior, or that societies should become tighter across all domains. This is particularly important because cultural norms and values are widely understood to shape innovation-related processes [12–20,60], potentially including knowledge creation, technological change, innovation performance, technology adoption, knowledge transfer, and diffusion. Tightness may support rapid convergence around behavioural rules, but it may also carry costs for openness, individual variation, creativity, dissent, and democratic deliberation. These features matter because scientific discovery and innovation often depend on experimentation, recombination, tolerance of variation, and the exploration of novel possibilities [8–11,21]. The same cultural characteristics that support rapid behavioural convergence in a crisis may, in other contexts, constrain the conditions under which scientific and technological advances are generated.

The findings therefore point to a broader knowledge-system paradox. Scientific capacity matters because societies need reliable knowledge, but knowledge does not protect populations unless it can be converted into coordinated action. Romer’s non-rivalry logic suggests that scientific ideas, once produced, can be reused widely at low marginal cost [9,55]. The example of oral rehydration therapy captures this logic especially clearly: once the simple scientific formula is known, it can be reproduced and applied across large populations at low marginal cost to prevent deaths [54]. Applied to COVID-19, this logic implies that knowledge about transmission, testing, treatment, suppression, mitigation, and behavioural interventions should, in principle, be scalable across countries [38,57–59]. However, the present findings suggest that non-rival knowledge does not automatically become non-rival protection. Scientific knowledge must be trusted, communicated, adapted, institutionalised, and behaviourally enacted before it improves population outcomes.

This distinction extends the Romer-Jones-Weitzman theoretical frame. Romer explains why ideas can scale and why their social value may exceed their private cost [10,55]. Weitzman’s recombinant logic explains why new ideas emerge from the exploration and recombination of existing possibilities [9,11]. Jones, however, cautions that the realised social return to knowledge may be weakened by duplication, congestion, absorptive limits, and a failure to effectively grow stocks of knowledge [56]. COVID-19 demonstrates the empirical importance of this qualification. Countries may possess large scientific systems, dense research networks, and extensive knowledge stocks, yet still fail to translate knowledge into population-level protection quickly enough. In a fast-moving crisis, scientific systems may produce large volumes of information without ensuring that this information is synthesised, trusted, communicated, and implemented at the speed required to reduce cases and deaths.

Cultural tightness may therefore operate less as an innovation mechanism than as an implementation mechanism. It may support the conversion of scientific guidance into behavioural regularity by reducing behavioural variance, lowering coordination costs, clarifying expected conduct, and increasing the likelihood that individuals comply with restrictions even when personal incentives favour deviation. This is consistent with the role of NPIs in pandemic response, the importance of individual responses to transmission guidance, and evidence that aggressive non-pharmaceutical interventions can shape pandemic trajectories [57–59,61]. In this interpretation, tightness does not necessarily increase the production of scientific knowledge. Rather, it may increase the probability that existing knowledge is enacted collectively when rapid coordination is required.

The paper therefore suggests a distinction between scientific capacity and knowledge-to-action capacity. Scientific capacity refers to the production, availability, and absorption of knowledge. Knowledge-to-action capacity refers to the ability of societies to convert knowledge into timely, trusted, coordinated, and behaviourally enacted response. Scientific capacity is related to scientometric output, cross-national knowledge networks, innovative capability, and the stock of prior related knowledge [22–32,39–53,60]. Knowledge-to-action capacity depends on institutional credibility, public communication, behavioural compliance, norm strength, trust, and the ability to coordinate collective action under uncertainty. The present findings suggest that disaster resilience depends on both. Scientific capacity may define what a society can know, but knowledge-to-action capacity shapes whether that knowledge changes behaviour at scale.

This distinction also helps explain why the relationship between culture and scientific capacity should not be reduced to a simple synergy or conflict. Looser cultural conditions may be more favourable to discovery perhaps because they permit experimentation, dissent, recombination, and variation [8–21]. Tighter cultural conditions may perhaps be more favourable to implementation when the problem requires rapid coordination around a narrow set of behavioural rules [1–7,57–61]. Pandemic response therefore reveals a division between the cultural conditions of discovery and the cultural conditions of implementation. Societies may need looseness to generate and recombine knowledge, but tightness to enact certain forms of life-saving guidance quickly. The challenge is not to choose tightness or looseness as a general social ideal, but to understand how societies can preserve openness for scientific discovery while enabling temporary behavioural coordination when collective action is urgent.

Mechanisms suggested by the additional literature help clarify why scientific capacity may not have shown a robust direct protective association in the multivariate models. Scientific knowledge might not translate directly into societal benefit as knowledge accumulation is itself collective, requiring collaborations and subject to constraints to collaboration [79], or prone to misinformation and may fail to translate into public beliefs and behaviours [80]. The findings therefore suggest that scientometric indicators of national scientific capacity should be complemented by measures of epistemic trust, public communication, policy translation, institutional credibility, and behavioural uptake.

Findings here seem broadly in line with other work suggesting tightness might aid compliance but constrain innovation [81]. The same literature also helps refine the interpretation of cultural tightness. Tightness may improve crisis response because it strengthens shared expectations, reduces behavioural variance, and makes deviation from public-health guidance more socially costly [82].

This interpretation is consistent with work suggesting that cultural tightness may aid coordination and compliance, while also being negatively associated with national innovativeness; conversely, cultural looseness, tolerance, and diversity of opinion may support innovation [82]. Related work using Hofstede’s dimensions suggests that cultures more oriented toward long-term change and more accepting of norm violations, reflected in lower uncertainty avoidance, tend to be more supportive of national innovation [83]. Other work on cultural dimensions and national innovativeness also suggests that cultural effects may operate through distinct micro-, meso-, and macro-level channels, rather than through a single uniform mechanism [84]. This may help explain why the present study finds the direct Gelfand tightness measure to be more robust than broader values-based indicators in predicting pre-vaccine COVID-19 outcomes.

System-justification theory further suggests that strong commitment to existing institutional arrangements can sometimes reinforce status quo bias, particularly under conditions of threat [84]. Such mechanisms may matter because the same social and institutional forces that support rapid compliance can, in other contexts, discourage dissent, experimentation, and adaptive problem-solving. These possible causal mechanisms are not directly tested here, and the findings should therefore be interpreted cautiously.

Nevertheless, the results are consistent with social and behavioural research arguing that COVID-19 response required large-scale behaviour change, and that behavioural science can help align public behaviour with epidemiological and public-health guidance [85]. This literature highlights the importance of social and cultural influences, science communication, leadership, threat perception, moral decision-making, and behavioural responses in pandemic control [85].

Related work on social norms and social identities further suggests that pandemic response required sustained mass behaviour change, often in ways that conflicted with existing social norms, and that public-health messaging may be more effective when it is aligned with salient group identities and supported by targeted interventions [86].

Taken together, these literatures strengthen the theoretical implication of the present findings. Cultural tightness may be valuable as a short-run implementation mechanism under acute collective threat, while looser or more pluralistic cultural conditions may remain important for long-run discovery, critique, adaptation, and innovation. Disaster resilience may therefore require institutional flexibility rather than permanent tightness. Societies need the ability to preserve openness for knowledge production while temporarily coordinating behaviour tightly when rapid collective action is required.

Overall, the findings contribute to debates on pandemic resilience, scientific capacity, and cultural tightness by showing that knowledge capacity and cultural coordination should be considered together. The results support the direct protective relevance of cultural tightness in the pre-vaccine phase, but do not support the stronger claim that tightness robustly moderates the association between scientific capacity and COVID-19 outcomes. The theoretical contribution is therefore not that tightness and science operate through a simple synergistic or antagonistic interaction. It is that scientific knowledge, even when non-rival and potentially life-saving, might have to pass through social, cultural, institutional, and behavioural conversion mechanisms before it can protect populations. This knowledge-to-action gap is central to understanding why scientific capacity alone may not have shielded societies from COVID-19, and why future research on disaster resilience should integrate scientometric, cultural, institutional, and behavioural explanations more closely.

## 6. Conclusions, limitations, and recommendations for further research

The objective of this paper was to test whether scientific capacity and cultural tightness were associated with lower COVID-19 cases and deaths during the pre-vaccine phase of the pandemic, and whether cultural tightness moderated the association between scientific capacity and these outcomes. The findings provide a cautious but important conclusion. Cultural tightness is consistently associated with lower cases and deaths, whereas scientific capacity does not show a robust direct protective association once cultural tightness and structural covariates are included. The interaction results are also limited. The evidence does not support the stronger expectation that tightness systematically weakens or strengthens the association between scientific capacity and COVID-19 outcomes.

The findings extend Romer’s theory of ideas by showing that the life-saving potential of non-rival knowledge might depend on the social conditions under which knowledge is implemented. Romer’s example of oral rehydration therapy illustrates the core logic. Once the formula combining water, sugar, and salts is discovered, the idea can be reproduced at very low marginal cost and deployed widely to prevent deaths from diarrhoeal disease. In this sense, codified scientific knowledge can scale human survival because its use by one person does not diminish its availability to others. Applied to COVID-19, the same logic suggests that scientific capacity should reduce cases and deaths by generating, validating, and diffusing knowledge about transmission, testing, treatment, behavioural guidance, and public-health protocols.

However, the results suggest that this Romerian logic is incomplete when applied to fast-moving societal crises. Scientific ideas may be non-rival, but their protective effects are not automatic. Knowledge must be interpreted, trusted, communicated, locally adapted, and converted into coordinated behaviour before it can reduce cases and deaths at population level. Scientific capacity may enlarge the feasible set of possible responses, but cultural and behavioural mechanisms influence whether those responses are implemented. The paper therefore identifies a knowledge-to-action gap between scientific capacity and realised human protection.

This interpretation is also consistent with Jones’s critique of simple knowledge-accumulation models. Jones highlights that the realised effects of knowledge may be weakened by duplication, congestion, absorptive limits, and failures in leveraging existing knowledge. The COVID-19 context provides a vivid example of this problem. Countries may have had large scientific systems, but the translation of scientific knowledge into public compliance, testing behaviour, treatment protocols, policy coherence, and behavioural restraint was not guaranteed. Scientific capacity was therefore not irrelevant; rather, its protective value appears to have depended on further institutional, cultural, and behavioural conversion processes.

The stronger association of Gelfand tightness with lower COVID-19 cases and deaths suggests that cultural tightness may operate as a social implementation mechanism. During the pre-vaccine phase, when response depended heavily on non-pharmaceutical interventions, the relevant constraint was not only whether scientific knowledge existed, but whether populations followed guidance consistently. Tightness may have reduced behavioural variance, clarified expected conduct, and increased compliance with social restrictions. The paper therefore contributes by showing that scientific ideas may save lives in principle, as Romer’s oral rehydration example demonstrates, but their realised effect may depend on the cultural and institutional capacity to convert ideas into coordinated action.

The findings also suggest a distinction between the cultural conditions of discovery and the cultural conditions of implementation. Romer and Weitzman explain why societies need openness, recombination, and ideational variety to generate life-saving knowledge. Jones explains why the social returns to knowledge may be weakened by congestion, duplication, and absorptive limits. COVID-19 shows that even when scientific knowledge exists, its protective value depends on whether societies can convert it into coordinated behaviour. Tightness may inhibit some forms of long-run ideation, but it may also increase short-run compliance with life-saving guidance. The paradox is therefore that cultural looseness may help produce the knowledge that saves lives, while cultural tightness may help enact that knowledge when rapid collective action is required.

These findings need to be interpreted in light of several limitations. First, the analysis is cross-sectional and should not be interpreted causally. The models identify associations between scientific capacity, cultural tightness, and pre-vaccine COVID-19 outcomes, but they cannot fully establish causal direction or rule out all omitted sources of confounding. Although cross-country cultural differences may be relatively stable over time, as suggested by evidence from the World Values Survey [66], this does not eliminate all concerns about endogeneity, measurement error, reporting differences, or unobserved institutional variation.

Second, the results are limited by the available data and by the measurement logic of the cultural indicators. Gelfand’s tightness measure and Uz’s looseness measure differ in construction, country coverage, and theoretical meaning. The findings therefore should not be read as proving that norm enforcement is causally superior to cultural homogeneity. They do suggest, however, that the cultural mechanism associated with pandemic response may be closer to norm strength, clarity, and enforceability than to cultural sameness alone. Future research should therefore distinguish more carefully between shared values, norm enforcement, institutional coercion, voluntary compliance, and trust.

Third, cultural tightness should not be treated as a general policy ideal. Whereas a growing body of literature highlights the importance of culture in explaining variation across countries and regions [1,3,5,8,21], tightness may have both benefits and costs. It may support collective behavioural coordination during acute crises, but may also constrain openness, dissent, creativity, experimentation, and democratic deliberation. Further research should therefore examine the conditions under which societies can preserve openness for discovery while enabling temporary coordination when collective action is urgently required.

A further limitation concerns causal direction and simultaneity. The analysis uses 2015–2017 country-level means for scientific capacity and the structural covariates in order to capture pre-pandemic conditions and reduce the risk that these variables are affected by COVID-19 cases, deaths, testing, or policy responses. Averaging across three pre-pandemic years also reduces sensitivity to single-year fluctuations. However, this does not eliminate all endogeneity concerns. Cultural tightness and related norms may reflect long-run historical processes, prior collective threats, institutional trajectories, and socialisation patterns, and the pandemic itself may also have strengthened norm enforcement, in-group cohesion, or behavioural conformity in some contexts. The cross-sectional design therefore cannot fully rule out reverse causality, omitted historical factors, or pandemic-induced changes in cultural response. Future research should use longitudinal designs, quasi-experimental approaches, and qualitative case studies to examine these mechanisms more directly.

Accordingly, further research should build on these findings using stronger causal designs. Variation in the timing of vaccine introductions, changes in public-health restrictions, and differences in institutional responses across countries may provide opportunities for natural experiments or quasi-experimental analysis. Case study and qualitative methods are also needed to identify the mechanisms through which scientific advice was translated into policy, public communication, trust, compliance, and behavioural change. Such work would help explain why some countries converted scientific capacity into effective response more successfully than others.

Further research should also develop more extensive datasets that capture knowledge-to-action capacity more directly. Scientometric measures of scientific output should be complemented by measures of real-time synthesis, policy translation, public communication, institutional trust, behavioural uptake, administrative capacity, and implementation quality. This is particularly important because the findings suggest that scientific capacity alone is not sufficient to protect societies from fast-moving threats. What seemingly matters is whether knowledge can be converted into timely, trusted, and coordinated action.

Notwithstanding these limitations, the paper contributes to research on scientific capacity, culture, and disaster resilience by showing that knowledge capacity and cultural coordination should be considered together. The catastrophic consequences of the COVID-19 pandemic [57,85,86] make this question especially important. Future pandemics, climate and environmental degradation, geopolitical conflict, and other systemic threats may similarly require both scientific knowledge and the capacity to translate that knowledge into collective action. The central implication is therefore clear: societies need not only the capacity to produce life-saving knowledge, but also the cultural and institutional capacity to act on it.

## Supporting information

S1 Dataset Final country-level analysis dataset in Microsoft Excel format. (CSV)

S2 Dataset Final country-level analysis dataset in Stata format. (DTA)

S1 File Stata analysis code used to reproduce the analyses reported in the article. (DO)

S1 Text README and data dictionary describing the variables, coding, data sources, and replication files. (TXT)
